# Denis Burkitt and the African lymphoma

**DOI:** 10.3332/ecancer.2009.159

**Published:** 2009-09-30

**Authors:** I Magrath

**Affiliations:** INCTR AISBL, 642 rue Engeland, 1180 Brussels, Belgium

## Abstract

Burkitt lymphoma has provided a model for the understanding of the epidemiology, the molecular abnormalities that induce tumours, and the treatment of other lymphomas. It is important to remember that the early phases of this work were conducted in Africa where today, unfortunately, the disease usually results in death because of limited resources, even though most children in more developed countries are cured. This must be changed. In addition, it is time to re-explore, with modern techniques, some of the questions that were raised some 50 years ago shortly after Burkitt’s first description, as well as new questions that can be asked only in the light of modern understanding of the immune system and the molecular basis of tumor development. The African lymphoma has taught us much, but there is a great deal still to be learned.

## Discovery of the tumour

**Figure 2:**
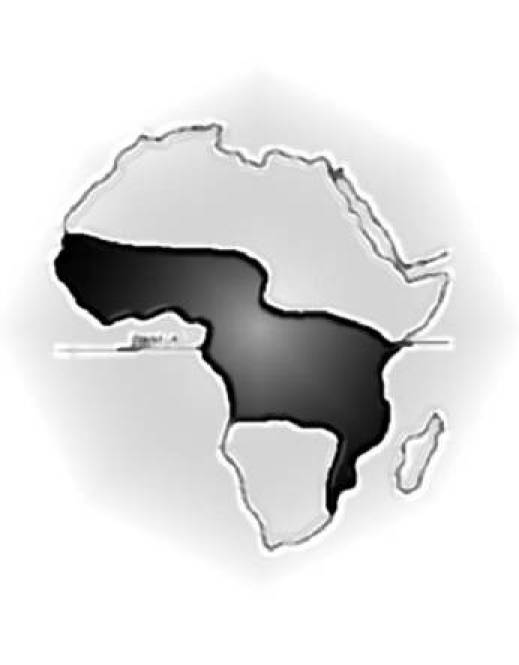
Map of Africa showing the ‘lymphoma belt’ in which Burkitt lymphoma occurs at high incidence. Lower incidence areas (e.g. highlands) within this zone are not shown.

Denis Parsons Burkitt was born in 1911 in Enniskillen, the picturesque county town of Fermanagh, now in Northern Ireland. ‘Enniskillen’ is derived from a Gaelic word meaning Ceithleann’s island, the town being situated on an island between two loughs (lakes) connected by the River Erne. According to Irish mythology, Ceithleann was the wife of Balor, the one-eyed king of a race of giants—a mythology that has echoes in Burkitt’s life. Sadly, at the age of 11, young Denis suffered an injury that led to the loss of an eye. Although this hampered his eyesight and, to a degree, his subsequent career as a surgeon, it had no effect on his insight. Burkitt doubtless inherited some of his observational skills from his father, James Parsons Burkitt, a civil engineer, but also an amateur ornithologist who was one of the first to use the technique of ringing or banding to recognize individual birds, allowing him to meticulously map their territories. James Burkitt’s maps must have impressed his elder son, who was later to map the distribution of the ‘African lymphoma’ (Figure 2).

Denis Burkitt attended the Portara Royal School at Enniskillen, one of five free schools founded by Royal Charter in 1608 by King James I.

In this respect, he followed in the footsteps of two Irish literary giants, Oscar Wilde and Samuel Beckett, who, like Burkitt, also continued their education at Trinity College, Dublin. Burkitt, without a clear idea of his future, had taken up engineering at Trinity College. He joined the University Christian Society, which gave new meaning to his life; he decided that his calling was to become a missionary. This, perhaps coupled to sharing a room with a medical student, led him to give up his engineering studies and instead take up medicine. After completing his studies, he decided to become a surgeon—somewhat surprising, perhaps, in view of his lack of binocular vision—and completed his basic training shortly before World War II.

Burkitt took a position as ship’s doctor before applying for a post in the colonial medical services. Unfortunately, he was turned down because he had only one eye. After other unsuccessful applications for an overseas posting, he decided to join the Army Medical Corps and, after working in England for a while, was sent to Africa, where he served in Somalia and Kenya. Once he spent leave in Uganda and visited the old Mengo Hospital, where the first missionary doctor to Africa, Sir Albert Cook, had worked, as well as the Mulago Teaching Hospital in Kampala, where he himself would later work. This experience, coupled to his evangelical zeal—and possibly the example of his uncle Roland, who practiced surgery in Nairobi—convinced him that he was destined to serve in Africa. In 1946, Burkitt again applied to the British Colonial Office for a post. This time he was accepted and was appointed to the position of District Medical Officer in Lira, a small town in the Northern Lango district of Uganda. While there he noted a high incidence of hydrocele, caused by mosquito-born filarial worms that block lymphatic vessels, and was able to show that the incidence was much higher in the eastern part of Lango (30% of men) than the western region (1%) [1]. This experience, too, must have sensitized him to geographic epidemiology while impressing upon him the important role of arthropod vectors in transmitting disease in Africa—a lesson that served him in good stead in the context of the African lymphoma.

Burkitt had been in Lira for only 18 months when he received a telegram summoning him to Mulago Hospital (Figure 5), where Ian McAdam, the only other formally trained surgeon in Uganda at the time, had become ill. McAdam, who subsequently became the head of the department of surgery in the University Hospital, was to become a strong supporter of Burkitt’s subsequent work.

**Figure 3:**
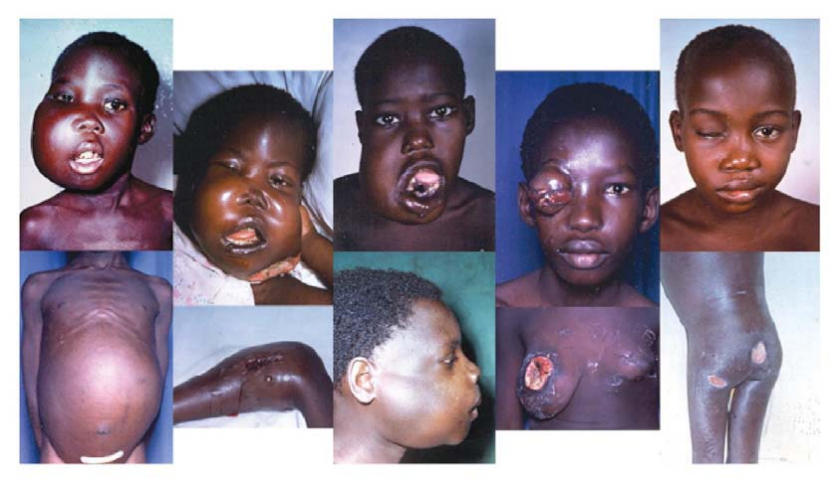
Children with Burkitt lymphoma showing multiple disease sites.

It was not until 1957 that Burkitt saw his first case of multiple jaw tumours (i.e. bilateral maxillae and mandibles) in a five-year-old boy he was asked to see in the children’s ward at Mulago Hospital by the pediatrician, Hugh Trowell. A biopsy report described the tumour as a ‘small round cell sarcoma’. Burkitt was unable to offer any advice on treatment, although the gross facial distortions caused by the jaw tumours made a big impression on him. Shortly afterwards he saw a second child, with tumour in all four jaw quadrants, during a regular visit to a hospital in Jinja, a small town situated where the river Nile flows out of Lake Victoria (one of the illusive sources of the Nile sought by the European explorers, Burton and Speke). This second child also had tumours in the abdomen. Like the first, his disease had been diagnosed as a small round cell sarcoma. The coincidence of seeing two children with jaw tumours in quick succession led Burkitt to examine the records of other patients seen in Mulago Hospital. He identified 29 other children who had presented with jaw tumours, although many, like the child in Jinja, had additional disease at other sites, including the orbit and abdomen, salivary glands, nervous system and elsewhere (Figure 3). Most of these tumours were diagnosed as small round cell tumours and variably reported, according to the sites of disease, as sarcoma, retinoblastoma, germinoblastoma, Ewing’s sarcoma, Wilms’ tumour or neuroblastoma.

Although several European pathologists working in Africa had observed the high incidence of jaw tumours, and of lymphomas in children with cancer many years before Burkitt saw his first case [2–5], Burkitt was the first to describe the clinical syndrome. He proposed that all the children with jaw tumours, regardless of other sites of disease, were probably suffering from the same disease. His first paper, entitled ‘A sarcoma involving the jaws of African Children’ was published in the British Journal of Surgery in 1958 [6]. This aroused little interest, since the disease offered limited scope for surgery. However, unknown to Burkitt, Gregory O’Conor and Jack Davies, pathologists also working at Mulago Hospital, were in the process of surveying the malignant tumours in children in the Mulago Hospital Registry that had been initiated seven years before. In fact, Davies had earlier observed that approximately half of the childhood tumours were derived from the ‘reticulo-endothelial system’ [7] and O’Conor and Davies’ more extensive review, which included children without jaw tumours, confirmed this [8]. It soon became clear that the tumour described by Burkitt was a lymphoma and, in 1961, Burkitt and O’Conor published additional papers in the journal *Cancer*, bringing it to the attention of cancer specialists [9,10]. Although it seemed initially that the tumour was confined to Africa, it was subsequently recognized that histologically identical lymphomas occur throughout the world, although at a much lower incidence [11–15] and with some differences at clinical and molecular levels from the African lymphoma [16].

The relationship of Burkitt lymphoma to acute lymphoblastic leukaemia (ALL) was frequently discussed since the latter disease was rarely seen in Africa. Dalldorf reported in 1962 that ALL, the commonest childhood malignancy in the USA, was the least common in East Africa, accounting for only 1–3% of childhood cancers in several published series [17]. ALL remains uncommon in equatorial Africa today—but that is another story.

## Discovery of Epstein-Barr virus

A few months after the publication of Burkitt’s first paper, Dr Oettle, a cancer specialist from South Africa, visited Kampala, and Burkitt was intrigued to learn from him that he had never seen children with a similar clinical syndrome in South Africa. This observation, influenced perhaps by his earlier work on hydrocele, led Burkitt to think about the geographical distribution of the tumour. Was it widespread throughout Africa, or confined to certain regions? Burkitt hung a map of Africa on the wall of his office and started to indicate places where children with jaw tumours had been seen. He sent 1000 brochures to government and mission hospitals throughout Africa and started to plot the ‘lymphoma belt’, as shown in Figure 2. By now, several research organizations were interested in the tumour, and Burkitt was given several grants, totalling £700, which enabled himself and two friends, Ted Williams and Cliff Nelson, both missionary doctors, to undertake a safari to define the southern limit of the high-incidence zone on the eastern side of Africa. Burkitt and his co-researchers set off from Kampala on 7 October 1961 in the 1954 Ford Station Wagon and returned ten weeks later, having visited some 57 hospitals in eight countries and travelled 10,000 miles. Although imprecise by today’s standards, the characteristic jaw tumours provided a powerful marker of the occurrence of the tumour, and Burkitt and his co-researchers were able to show that the ‘tumour belt’ extended to Lourenco Marques in southern Mozambique. Having defined in some detail the high-incidence regions, it appeared that in east Africa, at least, the barrier to the occurrence of Burkitt lymphoma was altitude [18,19]. Burkitt discussed this with Alexander Haddow, the director of the East African Virus Research Institute in Entebbe.

Haddow studied Burkitt’s map and pointed out that the altitude barrier occurred at 5000 ft at the equator (which passes through Uganda) and became progressively lower with the distance from the equator [20]. This strongly suggested that temperature rather than altitude constituted the true barrier, as had already been shown for a number of insect-born diseases, particularly those vectored by mosquitoes. Additional visits by air to Rwanda, Burundi, Kinshasa (Leopoldville at the time), Nigeria and Ghana confirmed the importance of altitude/temperature but also led to the recognition that low-lying areas where the tumour did not occur were arid regions, such as Kano in southern Nigeria. It seemed that the tumour was common in regions with temperatures that did not fall below 60°F, as long as there were at least 20 inches of rainfall per year—conditions required for mosquitoes to breed.

On seeing Burkitt’s new, improved map, Haddow confirmed that the distribution of the tumour conformed closely to the distribution of number of diseases known to be transmitted by insects, including trypanosomiasis (sleeping sickness), yellow fever and the recently recognized O’Nyong-Nyong. The findings seemed to be consistent with earlier research, particularly in the United States, that had demonstrated that malignancies in animals, particularly sarcomas and leukaemia, could be transmitted by viruses, although no one, at that time, had succeeded in identifying a human tumour virus.

Meanwhile, Burkitt had been receiving many invitations to speak. One of his lectures, given at the Middlesex Hospital in London in March 1961, was attended by a pathologist interested in virology, Michael Anthony Epstein. Impressed with the possibility that the lymphoma could be caused by a mosquito-vectored virus, Epstein approached Burkitt after the lecture and asked him to send some tumour samples to London in order to search by electron microscopy for virus particles in the tumour cells. Initial studies of tumour cells flown in from Uganda were negative, but Epstein and Barr succeeded in developing several continuously growing cell lines from the tumour cells. In the first of these, Epstein Barr-1 (EB1), Epstein *et al* were able to identify herpes-like virus particles in a small fraction of the cells. Unable to demonstrate reactivity with sera derived from patients known to be infected with other herpesviruses, it was clear that this was a previously undescribed virus and potentially the first human tumour virus. The report of their discovery was published in 1964 just a few years after the recognition of the unusual distribution of the African lymphoma [21].

It was soon shown that the virus associated with almost all African Burkitt lymphomas is ubiquitous, infecting almost the entire adult global population, although patients with African Burkitt lymphoma had a significantly higher antibody titre to the EBV virus capsid antigen (VCA) than controls [22]. The epidemiological relationship of EBV to Burkitt lymphoma was further demonstrated in a large serological study carried out by Geser *et al* in the West Nile district of Uganda. Starting in 1972, 42,000 children were bled between the ages of six months and two years and followed until March 1979 for the development of Burkitt lymphoma. In this time, 16 patients with Burkitt lymphoma were identified in whom a prior serum sample was available. Antibodies to the EBV VCA were also shown to be significantly higher in these patients in the pre-tumour sample [23]. This study demonstrated the very early infection with EBV in all of the children, showed that EBV infection can occur years before EBV-associated Burkitt lymphoma develops and was at least consistent with a causal role for the virus.

It has subsequently become clear that all tumour cells (and derived cell lines) in African Burkitt lymphoma contain multiple copies of the EBV genome, although the viral genome is present in only 1–10 per million circulating B lymphocytes (B lymphocytes, which are involved in antibody production, are the cell lineage in which EBV persists throughout life) [24,25]. This also suggests an important role for EBV, since African Burkitt lymphoma clearly occurs preferentially in the small fraction of EBV-containing B-cells in the body.

It was also demonstrated that EBV can ‘transform’ resting B cells into large proliferating cells, suggesting that EBV could be the driving force to the growth of Burkitt lymphoma. This proved to be wrong, however, for although EBV can ‘immortalize’ normal B lymphocytes *in vitro* and induce them to proliferate, in doing so it expresses nine viral genes (so-called latent genes) whereas only one of these genes, Epstein Barr virus nuclear antigen 1 (EBNA-1), along with some untranslated RNA molecules, are expressed in Burkitt lymphoma [24,25]. EBNA-1 is known now to be responsible for the replication of the viral genome, and for maintenance of the same number of genome copies in daughter cells. It does not drive proliferation. Actual virus is rarely produced in tumour cells because it is associated with cell disruption (lysis), allowing virus to escape and infect other cells or other people (an essential part of its life cycle). Thus, cell lines or tumours could not survive and the virus would not persist throughout life if all infected cells produced virus. We now have a detailed understanding of the structure and function of various EBV proteins, but the precise mechanism(s) whereby EBV contributes to the development of Burkitt lymphoma remains ill-defined [25].

## A role for malaria

The geographical studies carried out by Burkitt and others suggested to most that the African lymphoma was a rare manifestation of a common virus infection transmitted by mosquitoes. The subsequent discovery of EBV seemed to confirm this. However, it is now known that EBV is not transmitted by mosquitoes, but via saliva. In societies of low socio-economic status, there are many opportunities for saliva exchange, particularly mothers to infants. For example, in the absence of pureed baby foods, mothers often pre-chew the food they give to their infants during the weaning process. Thus, EBV infection, which in any case occurs throughout the world, cannot explain the high-frequency zone of Burkitt lymphoma in Africa. Interestingly, in the same year that EBV was discovered (1964), another explanation for the climatically determined distribution of Burkitt lymphoma was suggested—malaria. This possibility, first expressed in print by Gilbert Dalldorf, a microbiologist from Sloan-Kettering Institute in New York, had been heavily overshadowed by the vectored-virus hypothesis. By now, Burkitt’s enquiries had established that the tumour was also common in Papua New Guinea [26], and Dalldorf, in a detailed epidemiological study [27], pointed out that malaria was holoendemic in both equatorial Africa and New Guinea, and that repeated infections were nearly universal in the first year of life with variation in the intensity of infection being related to temperature and rainfall, which influence the breeding of mosquito vectors (*Anopheles* species) of malaria. He noted that in Kenya, the highest incidence rates of holoendemic malaria and the African lymphoma occur in identical areas—along the coast of the Indian Ocean and the shores of Lake Victoria. In 1965 Goma presented evidence based on extensive surveys in two districts in Uganda that Burkitt lymphoma tends to occur in communities which are very close (within a mile) to permanent or semi-permanent surface water, whether in the form of swamps or lake edges, where mosquitoes breed [28]. This doubtless explains why Burkitt lymphoma has a higher frequency in rural regions.

Burkitt and others began to explore the malaria (or ‘alternative’) hypothesis and made a number of tantalizing observations [29]. In the islands of Zanzibar and Pemba off the coast of Tanzania, and Leopoldville (now Kinshasa), intensive malarial eradication programs (directed at mosquitoes) had dramatically reduced the frequency of malaria (from 70% to less than 5%). In each case, in spite of the favourable climatic characteristics, Burkitt lymphoma had not been reported. Since then (in the case of Zanzibar, since 1964), the DDT-based eradication programs have been halted and malaria has returned to these regions—along with Burkitt lymphoma. In a meeting held by the International Union Against Cancer in 1967, Barnley pointed out that topography also influences the ecology of arthropods [30]. Steep river valley slopes, for example are not conducive to the persistence of temporary rain pools fully exposed to the heat of the sun, which are the preferred breeding places for *Anopheles gambiae* mosquitoes, and are generally devoid of malaria. He also stated that large bodies of fresh water (such as Lake Victoria) provide the major breeding grounds for *Anopheles funestus*. These findings were consistent with a role for a disease born by *anopheline* mosquitoes, but did not prove that malaria was the disease in question, although the positive relationship between the intensity of malarial infection and the occurrence of Burkitt lymphoma, a strong supporting argument, has been confirmed by a number of studies in both east and west Africa [31–34]. It has also been shown that in patients with acute malaria, the fraction of circulating lymphocytes containing EBV is increased in normal individuals, most probably as a result of impaired T-cell immunity, providing at least one mechanism whereby EBV and malaria can collaborate in the pathogenesis of Burkitt lymphoma [35–39].

In an attempt to demonstrate a role for malaria directly, Geser *et al* conducted clinical studies in the North Mara district of Tanzania designed to study the role of malaria directly [40]. In this region, active searching for cases of Burkitt lymphoma had been ongoing since 1970, permitting the measurement of incidence rates, along with surveys of malaria parasitemia and antibody levels). With this background information in hand, malaria prophylaxis (using a dose of chloroquine every two weeks) was initiated in all children aged one to ten years for a period of five years (1977–82). The incidence of malaria parasitemia initially fell to the lowest levels ever recorded, and the incidence of Burkitt lymphoma also dropped considerably (from four to less than one per 100,000 per year) and remained statistically significantly lower throughout the five-year period, beginning to rise only after the discontinuation of chloroquine prophylaxis (Table 1). However, the drop in incidence appeared to have begun prior to the administration of malaria prophylaxis, although this did not reach statistical significance. This could have been by chance alone, since the relatively small population of people in the North Mara lowlands where the trial took place—approximately 140,000 at the beginning of the study—is likely to result in a somewhat unstable annual incidence rate, although rates as low as those observed during the period of prophylaxis had not been seen in the course of a 13-year observation period before the start of the trial. In addition, it was found that there were defects in the system of distribution of chloroquine, which presumably accounted for the increase in parasitemia rates that began after only two years of prophylaxis. Thus, although these results are very suggestive of a role for malaria in the genesis of African Burkitt lymphoma, they are generally not considered to be definitive.

## Characteristic chromosomal translocations and the development of Burkitt lymphoma

In 1975, a characteristic chromosomal translocation (*t*8;14) was discovered by Zech *et al* in Burkitt lymphoma [41]. Subsequent molecular studies in the 1980s demonstrated that this and related ‘variant’ translocations resulted in the juxtaposition of an immunoglobulin gene (the genes responsible for the production of antibodies) to an oncogene called MYC—a gene heavily involved in a variety of critical cellular pathways including growth and programmed cell death (apoptosis). After many years of research, it is now believed that the ectopic (inappropriate) expression of MYC in B cells undergoing an immune response, most often caused by regulatory elements in the adjacent, translocated immunoglobulin gene, is the principle cause of the rapid, uncontrolled growth of Burkitt lymphoma cells. A number of reviews on this topic, which will not be discussed here, have been published [42–50].

Overall, the evidence suggests that at least one mosquito-borne disease, most probably malaria, predisposes to the development of Burkitt lymphoma. Malaria causes profound hyperplasia (overgrowth) of B lymphocytes—the cell lineage from which Burkitt lymphoma is derived.

It also results in an increase in the proportion of circulating memory B cells infected by EBV, strongly suggesting that the total body burden of EBV is also increased. This could result in an increased likelihood of the occurrence of the specific chromosomal translocations that are the proximate cause of Burkitt lymphoma. EBV probably also contributes directly to the development of the tumour, perhaps by inhibiting cell death by apoptosis, which would otherwise be induced by the inappropriate expression of MYC.

## Discovery of the response to chemotherapeutic agents

While the epidemiological questions raised by the observations made in Africa were intriguing, Burkitt and other clinicians in Africa were faced with the pragmatic problem of managing children with this disease.

Surgery was hardly an option—even on the rare occasions when tumour could be entirely removed, regrowth almost always occurred.

Radiotherapy was not then available in equatorial Africa (even now there are very few radiotherapy facilities in this region), but by the late 1950s a number of chemotherapeutic agents had become available and several were known to be particularly active in childhood ALL. It was clearly of considerable interest to know whether Burkitt lymphoma responded to chemotherapy. Joseph Burchenal, a pioneer chemotherapist working at the (then) Sloan-Kettering Institute for Cancer Research in New York, visited Burkitt in Uganda in 1960 and persuaded him to administer methotrexate, one of the drugs Burchenal had been working on in the context of ALL, to two children with Burkitt lymphoma. In both cases, a single dose induced dramatic tumour regression. This experience encouraged Burkitt to try other drugs, including cyclophosphamide.

Subsequently, investigators in Africa, including Burkitt in Uganda, Clifford in Kenya and Ngu in Nigeria, aided by western experts in chemotherapy, such as Oettgen and Burchenal from the Sloan-Kettering Institute for Cancer Research in New York, and David Galton from the Chester Beatty Institute in London, collaborated in documenting the response of Burkitt lymphoma to various chemotherapeutic drugs, often donated by the pharmaceutical industry (treatment studies are reviewed in [24,51,52]). Although these studies were not conducted in the disciplined way in which clinical trials are carried out today, and a significant fraction of patients was lost to follow-up, they led to the clear demonstration that Burkitt lymphoma was responsive to a broad range of chemotherapeutic agents. Burkitt, Clifford and Ngu reported some astonishing apparent cures with minimal therapy (several years of disease-free survival after only one or two cycles of therapy). In Burkitt’s series of 90 patients with jaw tumours treated at Mulago Hospital, Uganda, 74, or 82%, of the 90 patients had a good response.

Complete, durable remissions were observed with all three agents, although response depended upon the tumour size. Burkitt also noted that recurrent disease, whether at the same or different sites, did not occur after 11 months of remission, that is, patients free of disease at this time could be considered cured.

**Figure 5:**
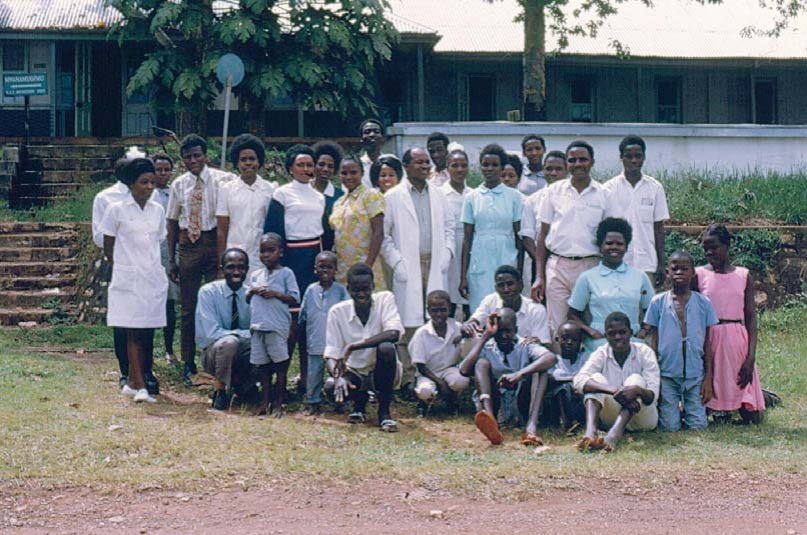
Staff of the Lymphoma Treatment Center in the Mulago Hospital complex in the early 1970s towards the end of the period of collaboration between Makerere University and the National Cancer Institute.

These early results laid the foundation for subsequent studies. Notable among these were clinical trials conducted in Uganda as part of a co-operative agreement between the National Cancer Institute of the USA and the University of Makerere in Kampala, Uganda (Figure 5). The emphasis eventually moved to drug combinations based on the most active drugs identified that led to a combination known as cyclophosphamide, vincristine and methotrexate (COM), which is the foundation of INCTR’s ongoing studies in the treatment of African Burkitt lymphoma (including AIDS-associated Burkitt lymphoma). In the United States and Europe, much more intensive drug combinations have since been developed, which have resulted in overall survival rates in the region of 90% [53].

Burkitt lymphoma has provided a model for the understanding of the epidemiology, the molecular abnormalities that induce tumours and the treatment of other lymphomas. It is important to remember that the early phases of this work were conducted in Africa where today, unfortunately, the disease usually results in death because of limited resources, even though most children in more developed countries are cured. This must be changed. In addition, it is time to re-explore, with modern techniques, some of the questions that were raised some 50 years ago shortly after Burkitt’s first description, as well as new questions that can be asked only in the light of modern understanding of the immune system and the molecular basis of tumour development.

The African lymphoma has taught us much, but there is a great deal still to be learned.

## Figures and Tables

**Figure 1:**
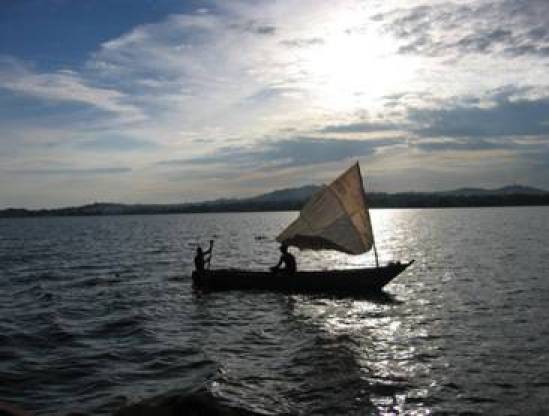
Lake Victoria, Uganda. The regions surrounding the lake are high-incidence regions for Burkitt lymphoma. Picture from Wikipedia Commons taken by D Luchetti.

**Figure 4:**
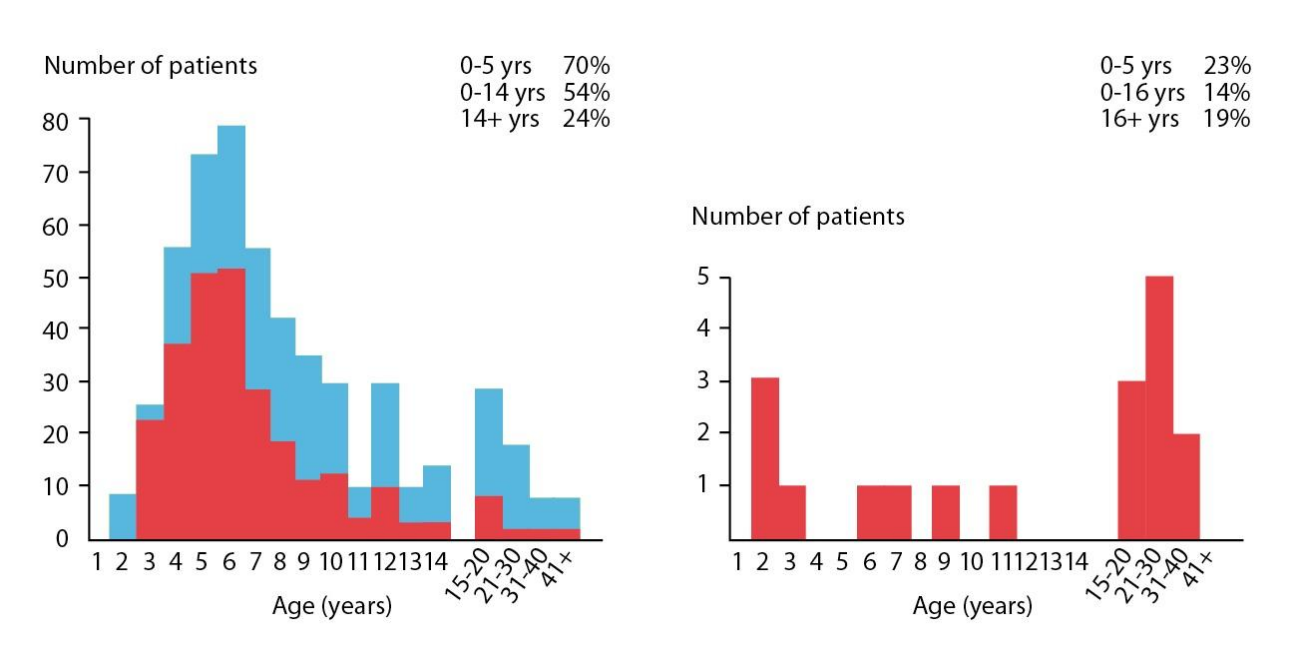
Age distribution of Burkitt lymphoma in Africa (a) and the United States (b). Jaw and orbital tumours are particularly common in young children in African Burkitt lymphoma (fraction of patients with jaw tumours is indicated by the red column in (a)) but not in Burkitt lymphoma in the United States.

**Table 1:**
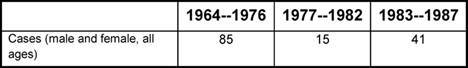
Cases of Burkitt lymphoma recorded in the North Mara District of Tanzania in three sequential time periods. Chloroquine prophylaxis was given in the middle period.
